# Nitrergic Signaling Mediation of Neurobehavioral Responses to Stress in Zebrafish

**DOI:** 10.1111/jnc.70254

**Published:** 2025-10-06

**Authors:** João Alphonse Apóstolo Heymbeeck, Eveline Bezerra de Sousa, Monica Lima‐Maximino, Antonio Pereira, Caio Maximino

**Affiliations:** ^1^ Programa de Pós‐Graduação Em Neurociências e Comportamento, Núcleo de Teoria e Pesquisa Do Comportamento Universidade Federal Do Pará Belém Brazil; ^2^ Laboratório de Neurofarmacologia e Biofísica Universidade Do Estado Do Pará – Campus VIII Marabá Brazil; ^3^ Programa de Pós‐Graduação Em Neurociências e Biologia Celular Instituto de Ciências Biológicas, Universidade Federal do Pará Belém Brazil; ^4^ Departamento de Patologia, Centro de Ciências Biológicas e da Saúde Universidade Do Estado Do Pará Belém Brazil; ^5^ Departamento de Morfologia e Ciências Fisiológicas, Centro de Ciências Biológicas e da Saúde Universidade Do Estado Do Pará Belém Brazil; ^6^ Faculdade de Engenharia Elétrica e Biomédica Instituto de Tecnologia, Universidad Federal Do Pará Belém Brazil; ^7^ Laboratório de Neurociências e Comportamento “Frederico Guilherme Graeff”, Faculdade de Psicologia Instituto de Estudos Em Saúde e Biológicas, Universidade Federal Do Sul e Sudeste Do Pará Belém Brazil

**Keywords:** gaseous transmitters, metaplasticity, post‐traumatic stress disorder, stress‐induced sensitization

## Abstract

Nitric oxide (NO) is a gaseous transmitter that is involved in the regulation of multiple behavioral processes in the brain. Here, we review the participation of this molecule in zebrafish neurobehavioral responses to stress. NO signaling pathways are considerably conserved in this species, and different pathways appear to be related to the complex regulation of behavior by this molecule. NO acts as a downstream integrator of responses to different upstream signals, including glutamate, serotonin, and inflammatory mediators; as a result, it participates in both anxiolytic and anxiogenic effects of drugs acting at different targets. There is considerable evidence for the participation of both a glutamate/NOS‐1 pathway and a KCNN/NOS‐2 pathway in long‐term behavioral sensitization to stress in zebrafish, suggesting that this molecule regulates metaplasticity in circuits that mediate defensive responses. Important questions remain unanswered, including the relationship between NO signaling, oxidative stress, and neuroinflammation, as well as the actual mechanisms of metaplasticity in the zebrafish brain.

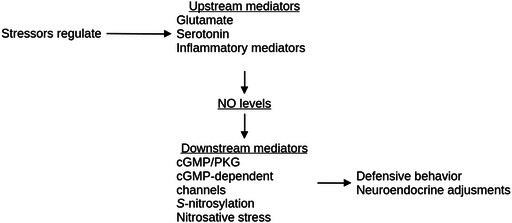

## Graphical abstract

Nitric oxide (NO) production is regulated by a plethora of upstream mediators, including glutamate, serotonin, and inflammatory mediators, at both neurons and non‐neuronal cells. These mediators are regulated by stressors, and their activity can increase or decrease NO production in a nitric oxide synthase (NOS) isoform‐dependent way. NO, in its turn, regulates cell and circuit activity through downstream mediators such as cGMP‐dependent protein kinase G (PKG), cGMP‐dependent channels, and mechanisms such as nitrosative stress and protein nitrosylation. The resulting state modulates defensive behavior (anxiety‐like and fear‐like behavior) and neuroendocrine adjustments.

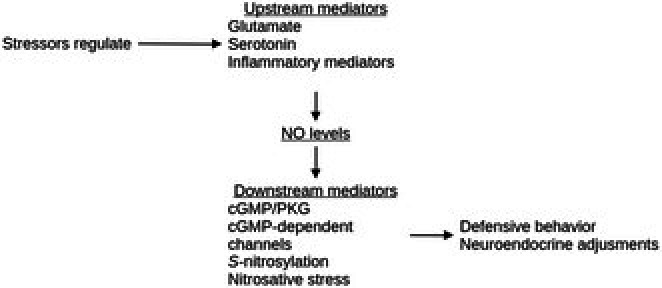

**Abbreviations**
5‐HT: Serotonin
5‐HTP: 5‐hydroxytryptophan
BSF: blue shortfin zebrafish
cAMP: cyclic adenosine monophosphate
CAS: conspecific alarm substance
cGMP: cyclic guanosine 3′,5′‐monophosphate
CNG: cyclic nucleotide‐gated channel
Dc: central zone of the dorsal telencephalic area
Dd: dorsal zone of the dorsal telencephalic area
Dl: lateral zone of the dorsal telencephalic area
Dm: medial zone of the dorsal telencephalic area
Dp: posterior zone of the dorsal telencephalic area
EBR: extreme Behavioral Response
EC: enzyme commission number
ENd: dorsal entopeduncular nucleus
ENv: ventral entopeduncular nucleus
ERK1/2: extracellular signal‐regulated kinase 1/2
FAD: flavin adenine dinucleotide
FMN: flavin mononucleotide
GTP: guanosine triphosphate
HCN: Hyperpolarization‐activated cyclic nucleotide–gated channel
IFN‐γ: interferon‐γ
IL: interleukin
IRF: interferon response factors
KCNN: calcium‐activated potassium channels
LC: locus coeruleus
LDP: long‐term depression
LDT: light/dark test
LFB: lateral forebrain bundle
L‐NAME: N^ω^‐Nitro‐L‐arginine methyl ester
L‐NOARG: N^ω^‐Nitro‐L‐arginine
LOF: longfin zebrafish
LOT: lateral olfactory tract
LTP: long‐term potentiation
MAPK: mitogen‐activated protein kinase
MBR: minimal behavioral response
MOT: medial olfactory tract
NADPH: nicotinamide adenine dinucleotide phosphate
NADPH‐d: nicotinamide adenine dinucleotide phosphate diaphorase
NF‐κB: nuclear factor kappa‐light‐chain‐enhancer of activated B cells
NMDA: *N*‐methyl‐D‐aspartate
NO: nitric oxide
NOHLA: Nω‐hydroxy‐L‐arginine
NOS: nitric oxide synthase
NOS‐1: nitric oxide synthase 1
NOS1AP: nitric oxide synthase 1 adaptor protein
NOS‐2: nitric oxide synthase 2
NOS‐3: nitric oxide synthase 3
NTT: novel tank test
ODQ: 1H‐[1,2,4]oxadiazolo[4,3,‐a] quinoxalin‐1‐one
PDE: phosphodiesterase
PKG: protein kinase
PPa: anterior parvocellular preoptic nucleus
PSD‐95: postsynaptic density protein 95
PTSD: post‐traumatic stress disorder
RNS: reactive nitrogen species
ROS: reactive oxygen species
SERT: serotonin transporter
sGC: soluble guanylate cyclase
SIS: stress‐induced sensitization
SNP: sodium nitroprusside
TNF‐α: tumor necrosis factor‐α
TrkB: tyrosine receptor kinase B
Vd: dorsal nucleus of the ventral telencephalic area
Vs: supracommissural nucleus of the ventral telencephalic area
Vv: ventral nucleus of the ventral telencephalic area

## 1 Introduction

Nitric oxide (NO) is a gaseous transmitter, a small family of mediators that include carbon monoxide and hydrogen sulfide (Donald 2016). Their strong lipophilicity and high solubility make these molecules intriguing transmitters, as they tend to freely permeate membranes and therefore do not necessarily act on cognate membrane receptors (Wang 2002). The diffusibility of NO also implies that it can act at multiple synapses, regulating different targets simultaneously (Ledo et al. 2004). Its role in mediating both the effects of glutamatergic signaling on neuroplasticity (Izquierdo et al. 2006; Medina and Izquierdo 1995) and neurotoxicity (Calabrese et al. 2007) positions it as an important mediator of brain functions and short‐ and long‐term adaptations to environmental challenges. Indeed, attention on the roles of NO in anxiety‐related neurobehavioral functions has soared in recent years (Pałasz et al. 2021)–especially in the long‐term sensitization of behavioral (dys)functions that are observed in trauma– and stressor‐related disorders, such as post‐traumatic stress disorder (PTSD) (Fronza et al. 2024; Oosthuizen et al. 2005). Indeed, a small study in Vietnam War veterans, including trauma‐exposed cases, showed that multiple *NOS1AP* and *NOS1* polymorphisms were associated with PTSD severity, stress, and resilience (Bruenig et al. 2017; Lawford et al. 2013), and the global arginine availability ratio, a marker of NO production, is reduced in war veterans with PTSD and is inversely related to symptom severity (Bersani et al. 2016).

Zebrafish ( *Danio rerio* Hamilton, 1822) are important organisms for modeling in biological psychiatry because they have well‐described defensive behaviors (Cachat et al. 2011; Egan et al. 2009), responsiveness to pharmacological treatments (Kysil et al. 2017; Maximino et al. 2014), and well‐defined behavioral and physiological changes when exposed to stressors (Champagne et al. 2010; Clark et al. 2011; Lima et al. 2016). There is significant translational potential in the use of zebrafish to study the role of NO in neurobehavioral stress responses, since, unlike rodents and as in humans, the response to stress is regulated by cortisol and the circadian cycle predicts a greater peak of activity during the day (Alsop and Vijayan 2009; Aluru and Vijayan 2009; Mommsen et al. 1999). In addition, its nitrergic system is sufficiently conserved in relation to other vertebrates (Holmqvist et al. 2000; Holmqvist et al. 2007; Virgili et al. 2001). In this review, we present current evidence on the complex regulation of neurobehavioral responses to stress in zebrafish. We begin by describing the nitrergic pathways in zebrafish in a comparative framework, and then discuss the roles of NO on anxiety‐like behavior and behavioral sensitization states. From these data, we then discuss NO as a downstream integrator of responses from different upstream signals, and end with a discussion on the roles of NO on metaplasticity.

## 2 Nitrergic Pathways in Mammals and Zebrafish

**FIGURE 1:**
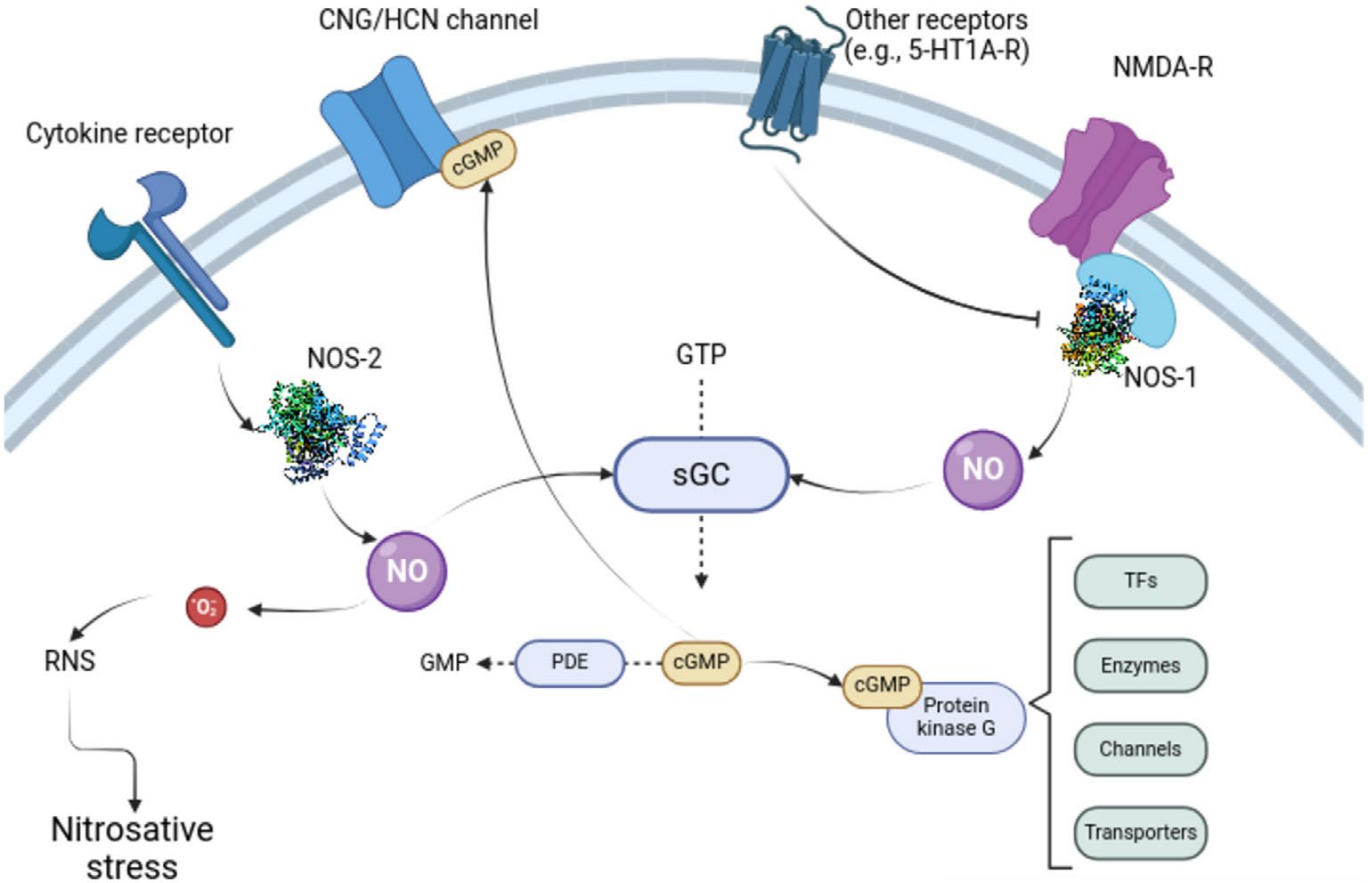
Multiple signaling pathways converge on nitric oxide (NO) production, and multiple downstream mediators are involved in its effects in vertebrates. Type 1 nitric oxide synthase (NOS‐1) is canonically coupled to the activation of glutamatergic NMDA receptors, but other receptors (e.g., serotonergic 5‐HT_1A_ and 5‐HT_1B_) regulate its activity, usually inhibiting NOS‐1 activity. NOS‐2, on the other hand, has its transcription regulated by the activation of cytokine receptors, as well as by other upstream signals (e.g., small‐conductance calcium‐activated potassium (KCNN) channels, not depicted). Both isozymes produce NO, with different regulation by co‐factors and different kinetics; NOS‐1 is calcium‐dependent and short‐lived, while NOS‐2 is calcium‐independent and long‐lived. NO activates soluble guanylate cyclase (sGC), synthesizing cyclic guanosine monophosphate (cGMP) from guanosine triphosphate (GTP). cGMP activates protein kinase G (PKG), which phosphorylates different targets (transcription factors [TFs]), enzymes, ion channels, and neurotransmitter transporters, among others, regulating the activity of these targets. cGMP can also activate cyclic nucleotide‐gated channels, including CNG and HCN channels, depolarizing or hyperpolarizing the neuron. Finally, NO can interact with reactive oxygen species such as superoxide (·O_2_^−^), producing reactive nitrogen species (RNS) such peroxynitrite, nitrogen dioxide, and dinitrogen trioxide, leading to nitrosative stress. Arrows (→) represent activation and/or upregulation of activity; box drawings (┤) represent inhibition and/or downregulation of activity.

Figure 1 summarizes nitrergic pathways. Nitric oxide is classically formed by the conversion of L‐arginine into L‐citrulline by one isoform of nitric oxide synthase (NOS; EC 1.14.13.39). This reaction runs by catalyzing a five‐electron oxidation of a guanidino nitrogen of L‐arginine; oxidation of L‐arginine to L‐citrulline occurs via two successive monooxygenation reactions producing N^ω^‐hydroxy‐L‐arginine (NOHLA) as an intermediate (Knowles and Moncada 1994). In mammals, three known NOS isoforms are encoded by different genes, *nos1*, *nos2*, and *nos3*; while NOS‐1 and NOS‐3 are constitutive, calcium‐dependent enzymes, NOS‐2 is inducible and calcium‐independent (Knowles and Moncada 1994). The evidence for a baseline NOS‐2 is elusive, but its induction by intracellular cascades associated with inflammatory cytokines, such as NF‐κB and IFR‐1, is well established (Green et al. 1994; Cinelli et al. 2020). In zebrafish, the NOS‐3 gene is not found (Lepiller et al. 2009); Lepiller et al. (2009) suggested that NOS‐3 diverged in mammals after two rounds of duplication, the first leading to the appearance of NOS‐2 in vertebrates, and the second leading to the appearance of NOS‐3 from NOS‐2 in the common ancestor between amphibians and mammals. Furthermore, in teleost fish, which passed through a further round of genome duplication, *nos2* was further derived into *nos2a* and *nos2b* (Table 1).

**TABLE 1:** Zebrafish genes that code for proteins in the nitric oxide (NO) signaling pathways and their orthologs found in humans.

Function	Protein	Zebrafish gene	ZDB ID	Human ortholog (Ensembl ID)
NO synthesis	NOS‐1	*nos1*	ZDB‐GENE‐001101‐1	*NOS1* (ENSG00000089250)
	NOS‐2	*nos2a*	ZDB‐GENE‐040305‐1	*NOS2* (ENSG00000007171)
		*nos2b*	ZDB‐GENE‐080916‐1	
	NOS‐3	Absent		*NOS3* (ENSG00000164867)
2nd messenger synthesis	sGC, subunit alpha 1	*gucy1a1*	ZDB‐GENE‐050417‐230	*GUCY1A1* (ENSG00000164116)
	sGC, subunit alpha 2	*gucy1a2*	ZDB‐GENE‐121023‐3	*GUCY1A2* (ENSG00000152402)
	sGC, subunit beta 1	*gucy1b1*	ZDB‐GENE‐090313‐160	*GUCY1B1* (ENSG00000061918)
	sGC, subunit beta 2	*gucy1b2*	ZDB‐GENE‐130530‐664	*GUCY1B2* (ENSG00000293412)
Effector proteins	PKG1	*prkg1a*	ZDB‐GENE‐040426‐1308	PRKG1 (ENSG00000185532)
		*prkg1b*	ZDB‐GENE‐080225‐40	
		*prkg1l*	ZDB‐GENE‐140422‐2	
	PKG2	*prkg2*	ZDB‐GENE‐060531‐80	*PRKG2* (ENSG00000138669)
	HCN1	*hcn1*	ZDB‐GENE‐110520‐1	*HCN1* (ENSG00000164588)
		*hcn1l*	ZDB‐GENE‐210225‐1	
	HCN2	*hcn2a*	ZDB‐GENE‐130530‐988	*HCN2* (ENSG00000099822)
		*hcn2b*	ZDB‐GENE‐030131‐8228	
	HCN3	*hcn3*	ZDB‐GENE‐060503‐193	Absent
	HCN4	*hcn4*	ZDB‐GENE‐050420‐360	*HCN4* (ENSG00000138622)
		*hcn4l*	ZDB‐GENE‐110411‐3	
	HCN5	*hcn5*	ZDB‐GENE‐081105‐169	Absent
	CNG, subunit alpha 1	*cnga1a*	ZDB‐GENE‐090312‐66	*CNGA1* (ENSG00000198515)
		*cnga1b*	ZDB‐GENE‐110408‐39	
	CNG, subunit alpha 2	*cnga2a*	ZDB‐GENE‐061005‐1	*CNGA2* (ENSG00000183862)
		*cnga2b*	ZDB‐GENE‐050307‐2	
	CNG, subunit alpha 3	*cnga3a*	ZDB‐GENE‐090611‐2	*CNGA3* (ENSG00000144191)
		*cnga3b*	ZDB‐GENE‐090312‐121	
	CNG, subunit alpha 4	*cnga4*	ZDB‐GENE‐070912‐292	*CNGA4* (ENSG00000132259)
	CNG, subunit beta 1	*cngb1a*	ZDB‐GENE‐050419‐164	*CNGB1* (ENSG00000070729)
		*cngb1b*	ZDB‐GENE‐090831‐4	
	CNG, subunit beta 3	*cngb3.1*	ZDB‐GENE‐090611‐1 LOC100334711	*CNGB3* (ENSG00000170289)
		*cngb3.2*	ZDB‐GENE‐091118‐102 LOC100334711	
Metabolism	PDE5	*pde5aa*	ZDB‐GENE‐100414‐2	*PDE5A* (ENSG00000138735)
		*pde5ab*	ZDB‐GENE‐060824‐4	
	PDE6	*pde6a*	ZDB‐GENE‐030616‐42	*PDE6A* (ENSG00000132915)
		*pde6b*	ZDB‐GENE‐090421‐2	*PDE6B* (ENSG00000133256)
		*pde6c*	ZDB‐GENE‐040426‐1664	*PDE6C* (ENSG00000095464)
		*pde6d*	ZDB‐GENE‐040718‐463	*PDE6D* (ENSG00000156973)
		*pde6ga*	ZDB‐GENE‐050522‐144	*PDE6G* (ENSG00000185527)
		*pde6gb*	ZDB‐GENE‐030904‐1	
		*pde6ha*	ZDB‐GENE‐040426‐1754	*PDE6H* (ENSG00000139053)
		*pde6hb*	ZDB‐GENE‐040426‐1754	
	PDE9	*pde9aa*	ZDB‐GENE‐130920‐1	*PDE9A* (ENSG00000160191)
		*pde9ab*	ZDB‐GENE‐210629‐1	
		*pde9ac*	ZDB‐GENE‐130530‐807	

Once a given NOS isozyme is activated, NO, as a gaseous transmitter, diffuses within and outside of the cell; this process depends not only on NO's diffusibility but also on the amount and rate at which it is generated, the duration of release from a source cell, and the rate (and compartmentalisation) of the NO reactions with O_2_ and other biological molecules, including reactive oxygen species (ROS) (Ledo et al. 2004). Thus, in cells that express NOS‐1, synthesis is relatively quick and short‐lived, and therefore diffusion is relatively limited; in that context, NO is likely to restrict its signal to a single synapse (or to a handful of close synapses), albeit its diffusion allows it to synchronize effects on many downstream mediators (Ledo et al. 2004). Thus, NOS‐1 activity is tightly regulated by cofactors such as calmodulin, NADPH, FMN, FAD, tetrahydrobiopterin, and heme (Groves and Wang 2000), as well as by the phosphorylation of serine/threonine residues by protein kinase A, C, and calcium/calmodulin‐dependent kinase (Bredt et al. 1992). In these cells, NOS‐1 is physically associated with the glutamate NMDA receptor via a complex of adaptor proteins, including PSD‐95 and NOS1AP, tightly coupling NMDA receptor activation and NO production (Li, Dustrude, et al. 2018).

There is some evidence that the NMDA/PSD‐95/NOS‐1 pathway in the mammalian limbic system is involved in regulating synaptic plasticity in fear consolidation and generalization. In goldfish, intratelencephalic injection of the non‐selective NOS inhibitor L‐NAME impaired active avoidance conditioning (Xu et al. 2009). In rats, after fear conditioning, a robust increase in the amygdala PSD‐95/NOS‐1 binding is observed; intra‐amygdalar infusion with ZL006, a compound that disrupts PSD‐95/NOS‐1 binding, attenuates fear memory (Li, Dustrude, et al. 2018). Treatment of amygdala slices with ZL006 also impaired long‐term potentiation (LTP), suggesting a participation in synaptic plasticity (Li, Dustrude, et al. 2018). In mice, contextual fear conditioning elicits a shift from PSD‐95/NOS‐1 to PSD‐95/TrkB association in the dorsal hippocampus, and microinjection of ZL006 in the dorsal hippocampus promotes contextual fear conditioning (Cai et al. 2018). Retrieval of contextual fear in a novel context at a remote time point (fear generalization, a process that is especially important for PTSD; Lopresto et al. 2016) up‐regulated PSD‐95/NOS‐1 coupling in the mouse anterior cingulate cortex, while systemic treatment with ZL006 before fear memory retrieval inhibits fear generalization (Qin et al. 2019). Thus, the NMDA/PSD‐95/NOS‐1 pathway appears to be fundamental in fear‐ and stress‐related plasticity processes that are important for PTSD.

In the case of inducible NOS isoforms (NOS‐2), a sequence variation at the hinge region allows it to bind tightly to calmodulin, and therefore the concentrations of calcium that are needed to activate the complex are much lower than other isoforms (Venema et al. 1996) – in fact, much lower than the typical cytoplasmic calcium concentrations, rendering inducible NOS isoforms effectively calcium‐independent (Cinelli et al. 2020). As a result, once NOS‐2 is induced, its production of NO is much higher for much longer than that mediated by other isoforms, and its diffusion will likely reach much further (Ledo et al. 2004). This is also relevant for differences in mechanism, since higher NO concentrations facilitates interaction with ROS and, as a consequence, to nitrosative stress (Cinelli et al. 2020; Ledo et al. 2004). The formation of reactive nitrogen species (RNS), such as the NO_2_ radical, peroxynitrite (ONOO^−^), nitrosoperoxycarbonate (ONOOCO_2_^−^), and dinitrogen trioxide (N_2_O_3_), represents a major mechanism for neurotoxicity (Pacher et al. 2007). NOS‐2 expression is typically induced by inflammatory stimuli, including the activation of cytokine receptors and downstream signaling via nuclear factor‐κB (NF‐κB) and interferon response factors (IRF) (Green et al. 1994) or the activation of calcium‐activated potassium (KCNN) channels, which are enriched in the microglia (Kaushal et al. 2007).

**FIGURE 2:**
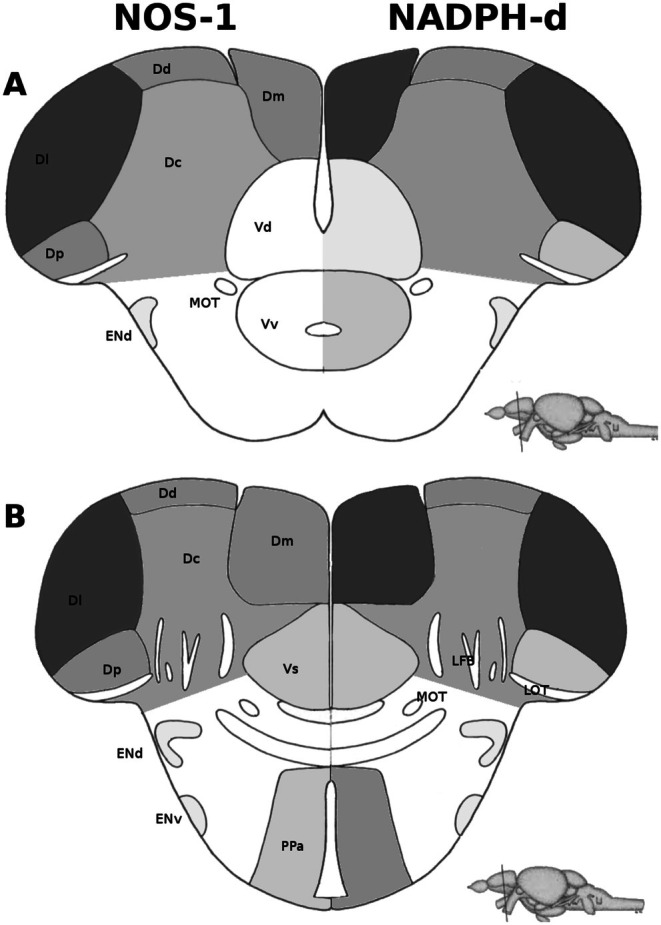
Distribution of nitric oxide synthase 1 (NOS‐1) expression and nicotinamide adenine dinucleotide phosphate diaphorase (NADPH‐d) activity in regions of the zebrafish telencephalon. Shading levels indicate levels of expression or activity. (A) shows a coronal section of the zebrafish telencephalon at the level of the commissura anterior, pars dorsalis. (B) shows a coronal section of the adult zebrafish telencephalon at the level of the commissura anterior, pars ventralis. Dc, central zone of the dorsal telencephalic area (homology not yet established); Dd, dorsal zone of the dorsal telencephalic area (isocortex homolog); Dl, lateral zone of the dorsal telencephalic area (hippocampus homolog); Dm, medial zone of the dorsal telencephalic area (pallial amygdala homolog); Dp, posterior zone of the dorsal telencephalic area (piriform cortex homolog); ENd, dorsal entopeduncular nucleus; ENv, ventral entopeduncular nucleus; LFB, lateral forebrain bundle; LOT, lateral olfactory tract; MOT, medial olfactory tract; PPa, anterior parvocellular preoptic nucleus; Vd, dorsal nucleus of the ventral telencephalic area (striatum homolog); Vs, supracommissural nucleus of the ventral telencephalic area (central/medial amygdala homolog); Vv, ventral nucleus of the ventral telencephalic area (septum homolog).

Information on the expression of NOSs proteins in the zebrafish brain is still scarce. Expression of *nos1* is first detectable in the brain at between 16 and 19 hpf in the hypothalamus and in a ventrorostral cell cluster of the forebrain (Holmqvist et al. 2004; Poon et al. 2003); after that period, *nos1* expression appears in the dorsorostral and ventrocaudal cell clusters of the forebrain, in the hindbrain and in the medulla (Holmqvist et al. 2004). In the embryonic brain, *nos1* expression is moderately associated with the proliferation zones of the brain (Poon et al. 2003). In the embryonic brain, *nos1* expression is associated with the expression of the protein tyrosine hydroxylase in the subventricular zone of the telencephalon, the dorsal hypothalamus, midbrain and hindbrain, including the locus coeruleus (LC) (Poon et al. 2003), suggesting that, in these regions, NO activity modulates catecholamine synthesis and/or release. *nos1* is also expressed in neurons of the embryonic and larval enteric nervous system (Holmqvist et al. 2004; Howard et al. 2021; Poon et al. 2003). In adults, *nos1* mRNA expression is found in the telencephalon, thalamus, hypothalamus (with strong expression in the posterior tuberal nucleus), optic tectum and rhombencephalon; in these latter regions, expression is weaker (Holmqvist et al. 2000). In the telencephalon, strong expression is found in the ventral subpallium, postcommissural nucleus, anterior parvocellular, and posterior zone of the pallium, with weaker expression in the medial and lateral zones of the pallium (Carreño Gutiérrez et al. 2020) (Figure 2). In both larvae and adults, *nos1* expression is higher near proliferation areas in the telencephalon, midbrain, and diencephalon, suggesting a role in regulating neurogenesis and gliogenesis (Holmqvist et al. 2000, 2004; Holmqvist et al. 2007). Overall, NOS‐1 appears to be widely expressed in the zebrafish brain, but its strongest expression levels are found in regions that mediate stress and defensive behavior (see do Carmo Silva et al. 2018, for a discussion of the zebrafish aversive brain system). Information regarding NOS‐2‐like proteins is even more scarce. PCR detects baseline levels of *nos2a* and *nos2b* in the adult brain, but the expression of these genes appears to be higher in the spleen, kidney, muscle, gut, ovary, and skin (Lepiller et al. 2009).

**FIGURE 3:**
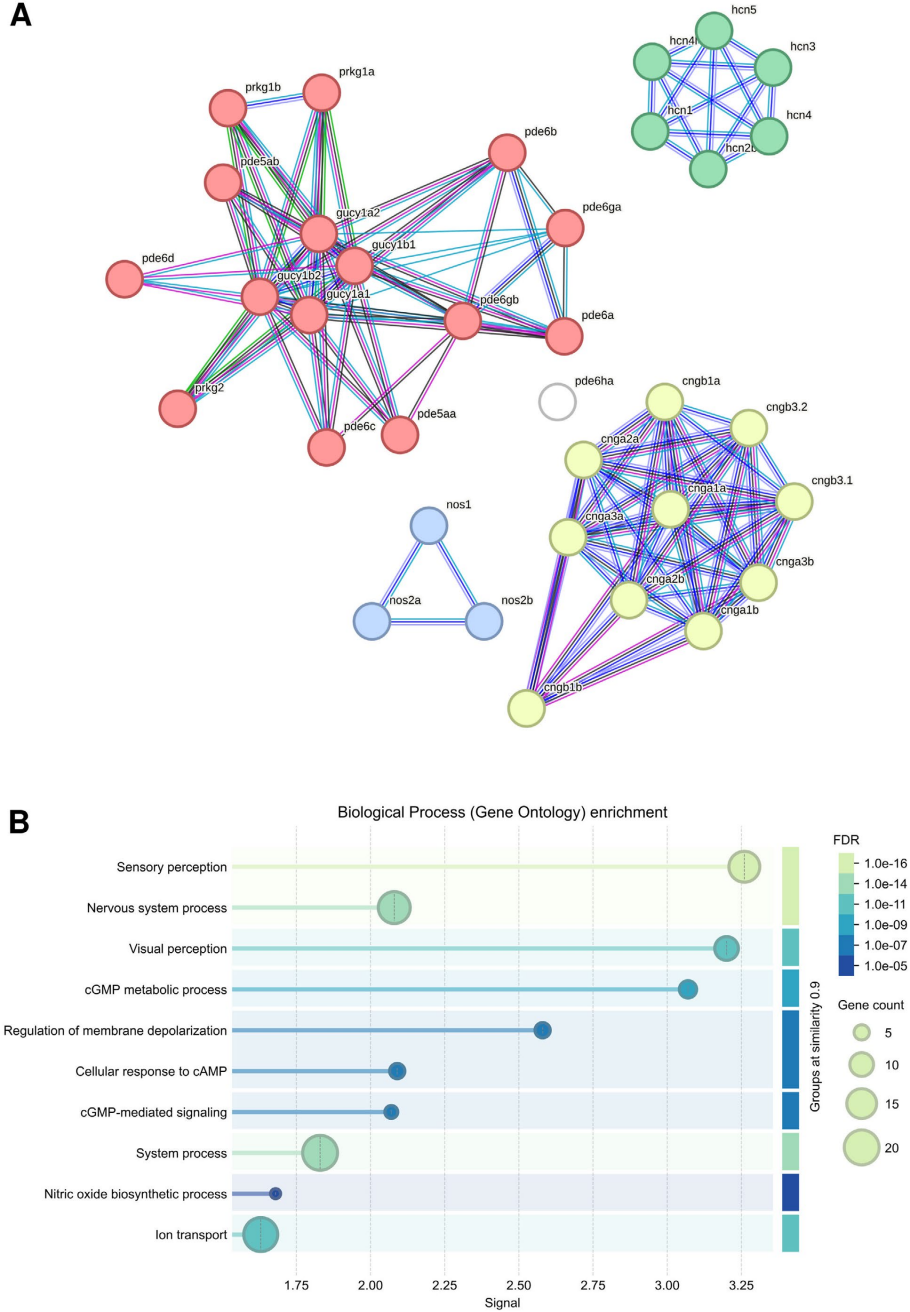
Protein interactions (A) and gene ontology‐based functions (B) for genes and proteins in the nitrergic signaling pathway found in zebrafish. In (A), nodes represent individual proteins, and edges represent protein–protein associations. Associations are meant to be specific and meaningful, i.e., proteins jointly contribute to a shared function; this does not necessarily mean they are physically binding to each other. Different node colors represent membership in different clusters, defined by *k‐*means clustering using data from the STRING database (https://string‐db.org/). The proteins from Table 1 were used as queries. The largest cluster, with nodes colored in pink, represent proteins involved in nucleotide metabolism, and includes phosphodiesterases, protein kinase G, and soluble guanylate cyclase. The second cluster, with nodes colored in yellow, represent proteins involved in intracellular cyclic nucleotide activated cation channel complex, including mainly subunits of the cyclic nucleotide gated channels (CNG). The third cluster, with nodes colored in green, represent proteins involved in up‐regulation of membrane depolarization, and includes subunits of the hyperpolarization‐activated cyclic nucleotide–gated channels (HCN). The last cluster, with nodes colored in blue, represent proteins involved in nitric oxide biosynthesis. Edges represent interactions based on known interactions from curated databases (light blue edges) and experimentally determined interactions (purple edges); and predicted interactions from gene neighborhood (green edges), gene co‐occurrence (dark blue edges), and co‐expression (black edges). In (B), functions of the proteins found in Table 1 are represented as gene ontology enrichment. Each line of the chart represents a specific function, with the length of the line representing enrichment signal strength and the size of the circle at the end of the line representing the number of genes from Table 1 that are involved in that function. The high enrichment in sensory perception/visual perception is due to the known functions of CNG and HCN channels in photoreceptors. Biological functions of these proteins are grouped by similarity (only when similarity scores were higher than 0.8), and the strength of shading represents false discovery rate (FDR). Functions were only included when FDR is lower than 0.05, and when they appear at least twice in the network.

The “canonical” intracellular target for NO is soluble guanylate cyclase (sGC; EC 4.6.1.2), a family of completely intracellular heterodimeric enzymes that are composed of one alpha and one heme‐binding beta subunit (Russwurm and Koesling 2002). As Figure 3 presents, most of the proteins involved in NO signaling in the zebrafish brain involve cGMP‐mediated signaling. In zebrafish, two alpha (*gucy1a1* and *gucy1a2*) and two beta (*gucy1b1* and *gucy1b2*) sGC subunits were described (Li, Zhou, et al. 2018; Table 1). sGCs catalyze the conversion of guanosine triphosphate (GTP) into cyclic guanosine 3′,5′‐monophosphate (cGMP), the canonical second messenger for the nitric oxide pathway. cGMP is degraded by cyclic nucleotide phosphodiesterases (PDEs); PDE5, PDE6, and PDE9 are cGMP‐specific while PDE1, PDE2, PDE3, PDE10, and PDE11 can hydrolyse both cyclic adenosine monophosphate (cAMP) and cGMP. Table 1 shows known zebrafish orthologs for cGMP‐specific PDE genes.

cGMP has at least three important targets in neurons and non‐neural cells: cyclic nucleotide‐gated (CNG) channels, hyperpolarization‐activated cyclic nucleotide–gated (HCN) channels, and protein kinase G (PKG) (Figure 1). A CNG channel consists of four subunits around a central pore; these subunits can be assembled from one alpha and one beta subunit (Table 1). Channels assembled from alpha 1 and alpha 3 subunits are primarily activated by cGMP, whereas the channels assembled from the alpha 2 subunit are equally sensitive to physiological concentrations of cAMP and cGMP (Kaupp and Seifert 2002). CNG channels are nonselective cation channels that poorly discriminate between alkali ions; at resting membrane potential, CNG channels conduct mixed inward currents carried by Na^+^ and Ca^2+^ (Kaupp and Seifert 2002). CNG channel functioning in photoreceptors and olfactory cilia has been widely demonstrated, but its roles in non‐sensory cells are still unclear; these proteins are expressed in the central nervous system by neuronal and glial cells and can influence neuronal excitability, neurotransmitter release, synaptic plasticity, growth cone guidance, synaptic bouton maturation, and neurogenesis (Charles et al. 2001; Podda et al. 2008, 2012, 2013; Togashi et al. 2008).

HCN channels are nonselective voltage‐gated cation channels that are activated by membrane hyperpolarization, are permeable to Na^+^ and K^+^, and are constitutively open at voltages near the resting membrane potential (Mishra and Narayanan 2025). These properties position HCN channels as important rhythm generators (“pacemaker cells”), allowing them to act as a negative feedback loop that suppresses the voltage response irrespective of which direction the input current flows in, effectively transforming neurons that express them into resonators (Mishra and Narayanan 2025). These characteristics also make HCN channels particularly interesting from the point of view of metaplasticity, since during long‐term potentiation their activity tends to increase, while it decreases during long‐term depression (Mishra and Narayanan 2025). Metaplasticity is defined as a change in the ability to induce subsequent synaptic plasticity such as long‐term potentiation or depression, without necessarily producing immediate changes in the efficacy of normal synaptic transmission (Abraham 2008; Abraham and Richter‐Levin 2018). As we will argue in Section 6, metaplasticity in limbic circuits is a potential mechanism through which these are sensitized by stress, resulting in long‐term behavioral effects.

The “canonical” pathway for NO, however, is the NO/cGMP/PKG pathway; PKGs are serine/threonine‐specific protein kinases that are activated by cGMP. In mammals, two isoforms are found, PKG‐I and PKG‐II; further complexity is added by the presence of two alternatively spliced exons at the N‐terminus of PKG‐I. In zebrafish, three PKG isoforms are found (Table 1). Substrates for PKG‐dependent phosphorylation include plasticity‐related molecules, ion channels, and transporters (Roy et al. 2021; Steiner et al. 2009; Zhang and Rudnick 2011; Zhu et al. 2004) (Figure 1). These substrates position PKG as an important effector in short‐ and long‐term plastic changes in brain function.

In addition to these mechanisms, NO also produces biological effects by *S*‐nitrosylation, the covalent attachment of a nitric oxide group to a cysteine thiol within a protein (Anand and Stamler 2012). Dysregulation of *S*‐nitrosylation has been implicated in the pathophysiology of different neuropsychiatric conditions, including Alzheimer's disease, Parkinson's disease, and stroke (Anand and Stamler 2012). While only limited evidence exists for the participation of *S*‐nitrosylation in mechanisms of stress and anxiety, in rats separated by anxiety levels in the elevated plus‐maze, high nitrosylation levels in the basolateral amygdala are observed in high‐anxiety animals, and treatment with diazepam or access to voluntary wheel running decreased both anxiety and nitrosylation levels (Shen et al. 2023). Sex differences are present in *S*‐nitrosylation, with female mice showing enrichment in *S*‐nitrosylation in synaptic processes in the cortex, while male mice show enrichment in cytoskeletal processes (Khaliulin et al. 2020).

## 3 Role of NO in Zebrafish Anxiety‐Like Behavior

Given the expression patterns of NO‐generating enzymes and upstream and downstream mediators in the zebrafish brain, it is no coincidence that NO appears to regulate anxiety‐like behavior in this species. The first evidence for a role in defensive behavior in this species (and in closely related teleost fish) came from experiments with active avoidance conditioning, which showed that injection of the non‐selective NOS inhibitor L‐NAME impaired the retention of conditioned avoidance when administered immediately after training (Xu et al. 2007). While avoidance is relevant to understanding active coping to threat (LeDoux et al. 2017), it is difficult to understand whether NO is involved in the processing of responses and habits or in more general memory processes. More evidence comes from studies using unconditioned behavior. In the novel tank test, chronic exposure to nitrite (NO_2_^−^) or nitrate (NO_3_^−^) for 31 days did not increase geotaxis, an effect that is probably explained by the fact that these treatments did not significantly increase NO_X_^−^ levels in the brain (García‐Jaramillo et al. 2020). These treatments, however, significantly decreased startle responses and produced an initial impairment in avoidance conditioning, although affected animals learned over repeated tests (García‐Jaramillo et al. 2020). Acute treatment with sodium nitroprusside (SNP), a NO donor, produced a biphasic dose–response pattern in adult zebrafish in the light/dark preference test, with lower doses decreasing scototaxis (anxiolytic‐like effect) and higher doses increasing it (anxiogenic‐like effect) (Herculano et al. 2015). *nos1* ^*−/−*^ zebrafish, generated by CRISPR/Cas9 technology aimed at exon 1 of the zebrafish *nos1* gene, displayed decreased shoaling, which could be indicative of an anxiogenic‐like role of NOS‐1, as well as higher expression of the dopamine transporter and higher tissue levels of dopamine (Penglee et al. 2021). Currently, however, it is not known whether this effect is associated with the defensive function of shoaling (and therefore with anxiety) or to an effect on social preference, since the roles of NO in social preference have not yet been investigated in zebrafish.

NO generation, in this case, is likely to be regulated by at least two mechanisms, a glutamatergic and a serotonergic one; treatment with either NMDA or the serotonin precursor 5‐HTP produces anxiogenic‐like effects that are blocked by pre‐treatment with L‐NAME (Herculano et al. 2015). Intracerebroventricular injection of NMDA in adult zebrafish increases scototaxis, while treatment with the nonselective NOS inhibitor N^ω^‐nitro‐L‐arginine (L‐NOARG) decreases it at a higher concentration and blocks the effects of NMDA treatment at a low concentration (Barbosa et al. 2012). Interestingly, treatment with either the 5‐HT1A receptor antagonist WAY 100635 or the 5‐HT1B receptor inverse agonist SB224,289 produces dose‐dependent *anxiolytic*‐like effects that were also blocked by L‐NAME (Herculano et al. 2015; Maximino, Lima, et al. 2015), suggesting that serotonin produces a tonic inhibition of NO production. While pharmacological tools, especially non‐selective inhibitors such as L‐NOARG and L‐NAME, make it difficult to define which isoform is associated with these effects, genetic research suggests that, at least in the case of the mediation of anxiety‐like behavior by the 5‐HT1A receptor, NOS‐1 is important. *nos1* ^*−/−*^ animals (generated by TALEN technology aimed at exon 1 of the *nos1* gene) show increased anxiety‐like behavior that is partially rescued by treatment with the 5‐HT1A receptor agonist 8‐OH‐DPAT (Carreño Gutiérrez et al. 2020). These effects are likely mediated by 5‐HT1A autoreceptors since *nos1* ^*−/−*^ animals show higher tissue levels of serotonin in the hindbrain (Carreño Gutiérrez et al. 2020). Moreover, *nos1* ^*−/−*^ zebrafish also show decreased monoamine oxidase activity and lower 5‐HT turnover in the telencephalon (Carreño Gutiérrez et al. 2020).

While serotonin can act as an upstream mediator of NO production, proteins in the 5‐HT pathway are also targets for this gaseous transmitter. The serotonin transporter (SERT) has been shown to interact with NOS‐1 in the mouse brain and in stable expression systems, leading to decreased membrane trafficking for the transporter and resulting in increased uptake capacity (Chanrion et al. 2007); interestingly, exposing HEK293 cells to 5‐HT (thus initiating uptake) increases cGMP production that is blocked by the NOS inhibitor L‐NAME and by serotonin uptake inhibitors (Chanrion et al. 2007). SERT also associates with PKG, and this association is necessary for increasing 5‐HT uptake by the PKG substrate 8‐Br‐cGMP (Steiner et al. 2009; Zhang and Rudnick 2011). Nitric oxide seems to increase 5‐HT uptake through a cGMP‐PKG‐dependent pathway (Miller and Hoffman 1994; Steiner et al. 2009; Zhang and Rudnick 2011; Zhu et al. 2004).

These pathways are also involved in the regulation of anxiety‐like behavior in zebrafish. Treatment with IB‐MECA, an agonist at adenosine A3 receptors, produces anxiolytic‐like effects in both the light/dark test and the novel tank test; these effects are mediated by serotonin, as IB‐MECA also decreased extracellular 5‐HT levels ex vivo and increased 5‐HT uptake from zebrafish synaptosomes in vitro (Maximino, Gemaque, et al. 2015). The effects of IB‐MECA on 5‐HT uptake were blocked by L‐NAME and potentiated by 8‐bromo‐cGMP, demonstrating the mediation of NO in this mechanism; importantly, the serotonin reuptake inhibitor fluoxetine, the calcium channel blocker verapamil, and the NOS inhibitor L‐NAME blocked the behavioral effects of IB‐MECA, demonstrating a mechanism of interplay between adenosine A3 receptors, nitric oxide, and serotonin uptake in the control of defensive behavior in zebrafish (Maximino, Gemaque, et al. 2015). At present, it is impossible to know which isoform mediates these effects.

While the specific targets of cGMP in the control of zebrafish defensive behavior are unknown, there is some evidence that this molecule is important in the regulation of anxiety‐like responses. Treatment of zebrafish larvae with the non‐selective PDE inhibitor rolipram induced thigmotaxis that was dependent on the ability of the fish to detect differences in light conditions (Lundegaard et al. 2015); however, this effect was mimicked by both forskolin, a small molecule that activates adenylate cyclase (and therefore cyclic adenosine monophosphate production), and by the PDE4 selective inhibitor rolipram, suggesting that it is mediated by cAMP and not cGMP. In goldfish ( *Carassius auratus* ), a closely related cyprinid, telencephalic cGMP is crucial for the consolidation of aversive conditioning (Xu et al. 2009). As discussed below, treatment with the sGC inhibitor 1H‐[1,2,4]oxadiazolo[4,3,‐a]quinoxalin‐1‐one (ODQ) blocks the development of stress‐induced sensitization in adult animals exposed to conspecific alarm substance, suggesting a role for cGMP in stress‐related metaplasticity (de Sousa et al. 2024).

## 4 Role of NO in Sensitization Processes

Response sensitization can be defined as the sustained manifestation of behavioral and physiological adaptations following exposure to a specific stressor. This process requires an “incubation” period during which both associative and non‐associative mechanisms can be implicated (de Sousa et al. 2024; Stam 2007a, 2007b). NO plays a critical role in neural plasticity due to its involvement in the cellular mechanisms underlying learning and memory processes, which are associated with the observed adaptations (Bredt and Snyder 1992). In this regard, conditions such as post‐traumatic stress disorder (PTSD) and alcohol/drug withdrawal are phenomena in which these adaptations and the NO action appear to be closely linked (Elvig et al. 2021; Karanikas et al. 2021).

### 4.1 Stress‐Induced Sensitization

In PTSD, exposure to an extremely stressful and life‐threatening event triggers a state of hyper‐responsiveness to trauma‐associated stimuli, reflecting the sensitization of a set of physiological and behavioral responses in the organism (Bailey et al. 2013). NO appears to be involved in the consolidation phase of sensitization of behavioral adjustments related to stress induced by conspecific alarm substance (CAS) in zebrafish (de Sousa et al. 2024; Lima et al. 2015). The model used in this context is based on time‐dependent sensitization, which provides validity for studies related to PTSD, enabling the investigation of neurobiological and behavioral mechanisms associated with the disorder (Lima et al. 2016).

Anxiety‐like behavior sensitization 24 h after CAS exposure is observed in the longfin (LOF) and blue‐shortfin (BSF) phenotypes, though differences exist between these wild‐type zebrafish populations when assessed in the light/dark test (LDT) and novel tank test (NTT). LOF animals appear more susceptible to sensitization than BSF animals, reflecting a baseline anxiety. The observation that control LOF animals also exhibit such differences supports this proposition (Lima et al. 2016). These findings suggest the validity of stress‐induced sensitization (SIS) for PTSD modeling, as anxiety sensitivity appears to predict the development of disorder symptoms (Fedroff et al. 2000). Another important factor for PTSD modeling relates to observations that LOF animals are differentially affected by sensitization: approximately 25% exhibit Extreme Behavioral Response (EBR), with increased erratic swimming compared to unexposed animals and stronger effects on scototaxis and thigmotaxis, while about 20% show little to no sensitization (Minimal Behavioral Response, MBR), percentages consistent with PTSD epidemiology, in which 15%–35% of individuals exposed to a traumatic event meet diagnostic criteria (Goswami et al. 2013; Yehuda and LeDoux 2007). MBR animals appear “resilient” to CAS‐induced stress, as risk assessment was higher in both exposed subgroups compared to unexposed animals (Lima et al. 2016).

In the BSF phenotype, CAS exposure sensitizes geotaxis, erratic swimming, and thrashing in the NTT (Lima et al. 2016). Prolonged predator exposure (24 h or 72 h) also increases geotaxis and erratic swimming events, interpreted as PTSD‐like behavior (Stewart et al. 2014). In the LDT, sensitization of scototaxis, risk assessment, erratic swimming, and thigmotaxis can be observed (Lima et al. 2016). Increased risk assessment and thigmotaxis are only observed 24 h post‐exposure, whereas erratic swimming effects appear diminished compared to immediate post‐exposure assessment. Freezing, however, increases only immediately after exposure (Lima et al. 2016). Discrepancies exist in geotaxis duration data across studies conducted under similar conditions, as this behavior does not appear consistently altered in all of them (Cachat et al. 2010; Lima et al. 2016; Quadros et al. 2016), possibly due to methodological differences.

CAS exposure appears to sensitize behavioral responses to heterotypic stressors, as the fear‐like state induced by CAS is not *sustained* but sensitizes behaviors manifested in response to the aversive properties of the NTT and LDT. It is thus proposed that the incubation period sensitizes responses to heterotypic stressors (Lima et al. 2016). These parallels suggest that stress sensitization can model some aspects of PTSD, including the main behavioral endpoints and the processes through which stress is “incubated” resulting in prolonged over‐reactivity to mildly stressful conditions. Caution must be taken, however, in not over‐interpreting these results; PTSD is a very complex human disorder, involving not only stress incubation but a plethora of psychosocial aspects that do not readily cross‐translate to other organisms (Borghans and Homberg 2015). Nonetheless, if caution is exerted in extrapolation, zebrafish stress‐induced sensitization models can be useful to produce novel hypotheses on the roles of mediators such as NO in neurobehavioral phenomena that are relevant for PTSD.

There is a series of evidence that suggests that this sensitization is mediated by nitrergic signaling that occurs in a short period after CAS exposure, as well as the sustained production of NO at least up to 24 h after stress. In zebrafish, forebrain glutamate levels rise immediately and 30 min following exposure to CAS, and nitrite levels increase immediately after stress, 30 min after stress, 90 min after stress, and 24 h after stress (de Sousa et al. 2024). Treatment with L‐NAME, a non‐selective NOS inhibitor, reverses the effects on erratic swimming and thigmotaxis 90 min after stress, but not 30 min after stress, whereas an opposite pattern is observed for scototaxis (Lima et al. 2015). Consistent with the enzyme specificity of nitrite production in both time intervals, blocking NOS‐1 with 7‐NI 30 min after stress prevents stress‐induced sensitization, while aminoguanidine treatment 90 min after stress prevents the phenomenon (de Sousa et al. 2024). Interestingly, nitrite levels are also increased in the head kidney (where cortisol‐producing cells are located in zebrafish) 30 min and 90 min after CAS exposure, but aminoguanidine was not able to block this effect (de Sousa et al. 2024), suggesting the participation of other NOS isozymes. Nonetheless, both baseline and acute stress‐elicited cortisol levels are not affected in *nos1* ^*−/−*^ animals (Carreño Gutiérrez et al. 2020), and therefore how NO regulates neuroendocrine responses to stress in zebrafish is still an outstanding question (Table 2). The effects of 7‐NI and aminoguanidine on forebrain nitrite suggest that brain NOS‐1 is involved in the first stages of stress‐induced sensitization in zebrafish, while NOS‐2 is involved in later stages. On the other hand, treatment with the NOS‐2 inhibitor aminoguanidine blocks CAS‐elicited increases in forebrain nitrite that are observed 90 min after stress, but not 30 min after stress, suggesting that NOS‐2 is recruited in later periods of the incubation period (de Sousa et al. 2024); aminoguanidine also blocks stress‐induced sensitization when administered 90 min after stress, confirming the role of this enzyme in SIS (de Sousa et al. 2024). Interestingly, blocking calcium‐activated potassium (KCNN) channels 90 min after stress also prevents stress‐induced sensitization (de Sousa et al. 2024), suggesting that these upstream activators of NOS‐2, which are enriched in the microglia (Kaushal et al. 2007), are partially responsible for this mechanism. sGC inhibition with ODQ prevents the sensitization of behavioral responses in both time windows associated with NO production by NOS‐1 and NOS‐2 isoforms, corresponding to treatments administered 30 and 90 min after the stressor, respectively (de Sousa et al. 2024). These results indicate that downstream cGMP signaling plays a critical role in the sensitization mechanisms. Additionally, CNG channels appear to be involved in sensitization during the second time window, potentially modulated by either cAMP or cGMP (de Sousa et al. 2024). The picture that emerges for the role of NO in SIS is that fear‐eliciting stressful stimuli first activate telencephalic circuits via the NMDA/PSD‐95/NOS‐1 pathway, initiating a process of metaplasticity. A second wave of NO‐dependent sensitization occurs via the KCNN/NOS‐2 pathway, and it extends NO production for a much longer period (at least 24 h after stress, when behavioral alterations are observed in the stress‐induced sensitization model). During the first moments of the incubation period, NO effects appear to be mediated by the cGMP/CNG channel pathway, but the importance of NOS‐2 in sustained NO production also suggests the participation of nitrosative stress.

**TABLE 2:** Outstanding questions still open in relation to the roles of NOergic signaling in zebrafish neurobehavioral responses to stress.

Outstanding questions
What (if any) are the differences in nitrergic signaling across sex?
What is the relationship between NO and neuroinflammation?
What is the actual role of metaplasticity in stress‐induced sensitization?
What is the possible intersection between neuroinflammation and neuroplasticity in zebrafish stress responses?
What, if any, is the role of NO in regulating neuroendocrine responses to stress in this species?

The participation of the KCNN/NOS‐2 pathway seems to implicate neuroglia. While it is currently unknown whether these effects occur in the microglia, the KCNN channels are intimately linked to microglia activation in vitro (Kaushal et al. 2007). Indeed, the NOS‐2 pathway is classically associated with neuroinflammation (Munhoz et al. 2008). Exposure of zebrafish to a combination of stressors (vortexing in cold water with lighting, exposure to shallow water, and restraint) produces stress‐induced sensitization 1 week after exposure, an effect that is accompanied by increased mRNA expression of genes in the brain encoding the glucocorticoid receptor, neurotrophin BDNF and its receptors, microglial markers, astrocytal markers, as well as pro‐inflammatory cytokines IL‐6, IL‐1β, IFN‐γ, and TNF‐α (Yang et al. 2020). Combined with results with NOS‐2 and KCNN channel inhibitors (de Sousa et al. 2024), this raises the interesting question on the roles of the KCNN/NOS‐2 pathway in producing these changes in neuroglia, as well as in the regulation of neuroinflammation in the zebrafish brain. Since cytokine signaling can also activate NOS‐2, it is also possible that a cytokine/NF‐κB (or IRF‐1)/NOS‐2 pathway is also involved in stress‐induced sensitization. This hypothetical mechanism remains an outstanding question in the field (Table 2).

An important addition to zebrafish models in understanding the roles of NO in sensitization processes would be the analysis of sex differences. The target disorder, PTSD, shows differences in prevalence between cisgender men and women (Olff 2017). While gender‐based violence explains most of these differences in humans, neurobiological substrates might also differ. Moreover, there is some evidence for differences in NO signaling (e.g., *S‐*nitrosylation; Khaliulin et al. 2020) in male and female brains, which could suggest different roles for this complex pathway across sexes. There is some evidence for sex differences in stress responsiveness in zebrafish (Genario et al. 2020) which could help understand part of these sex and gender effects in humans as well. This also remains an outstanding question in the field (Table 2).

### 4.2 Alcohol/Drug Withdrawal

The NOS‐2 isoform appears to be implicated in stress sensitization mechanisms, given that its activity is calcium‐independent and can be induced by sustained psychological stress. In this process, NO production via NOS‐2 is continuous until the enzyme is degraded, provided substrate is available (Cinelli et al. 2020). Dysphoric states resulting from the abrupt discontinuation of drug and/or alcohol use appear to exemplify this phenomenon, which is relevant for understanding symptoms associated with chronic substance abuse (Chaves et al. 2020).

Adult zebrafish subjected to 20 min/day ethanol treatment for 8 days, followed by 7 days of withdrawal, exhibit increased anxiety‐like behavior in the NTT and increased nitrite levels in the brain, effects that were blocked by administration of aminoguanidine (Chaves et al. 2020). The anxiety‐like responses observed after withdrawal include increased geotaxis and freezing (Chaves et al. 2018; Chaves et al. 2020). Literature discrepancies exist regarding the sensitization of erratic swimming (Chaves et al. 2020; Tran and Gerlai 2013), which may be attributed to differences in exposure duration and ethanol concentration (Chaves et al. 2018). The fact that animals treated with aminoguanidine on day 8 of ethanol exposure do not exhibit sensitized behavioral responses after the 7‐day withdrawal period suggests a strong involvement of NOS‐2 in the behavioral adaptations manifested as anxiety‐like responses in untreated animals following withdrawal (Chaves et al. 2020).

This role of NOS‐2 raises the suspicion that oxidative and nitrosative stress could be implicated in the behavioral sensitization caused by ethanol withdrawal. Indeed, the increase in anxiety‐like behavior resulting from ethanol withdrawal in adult zebrafish is associated with reduced brain activity of catalase, an antioxidant enzyme (Chaves et al. 2018). This effect is blocked by N‐acetylcysteine, an antioxidant agent (Mocelin et al. 2019). Thus, part of the observed oxidative imbalance appears to be related to nitrosative stress (Chaves et al. 2020). Zebrafish possess two distinct genes responsible for NOS‐2 expression—*nos2a* and *nos2b*—both of which are expressed in the central nervous system and are upregulated following inflammatory stimuli, suggesting their potential involvement in this response (Lepiller et al. 2009). The finding that chronic ethanol exposure induces oxidative stress without increasing nitrite levels suggests that elevated nitrite is specifically associated with withdrawal in zebrafish (Agostini et al. 2018).

## 5 NO As a Downstream Integrator

The interactions between NO and 5‐HT point to a complex interplay between NO and 5‐HT in the regulation of anxiety‐like behavior in zebrafish. Moreover, the results from traumatic stress models commented above also suggest that NO production can be elicited by other mechanisms, including the activation of receptors that are likely to be found in microglia and subsequent induction of NOS‐2 activity. Thus, NO appears to be a downstream integrator of responses that can decrease or increase defensive behavior, depending on the environmental and behavioral context and, as a consequence, the neurochemical milieu in different brain regions.

**FIGURE 4:**
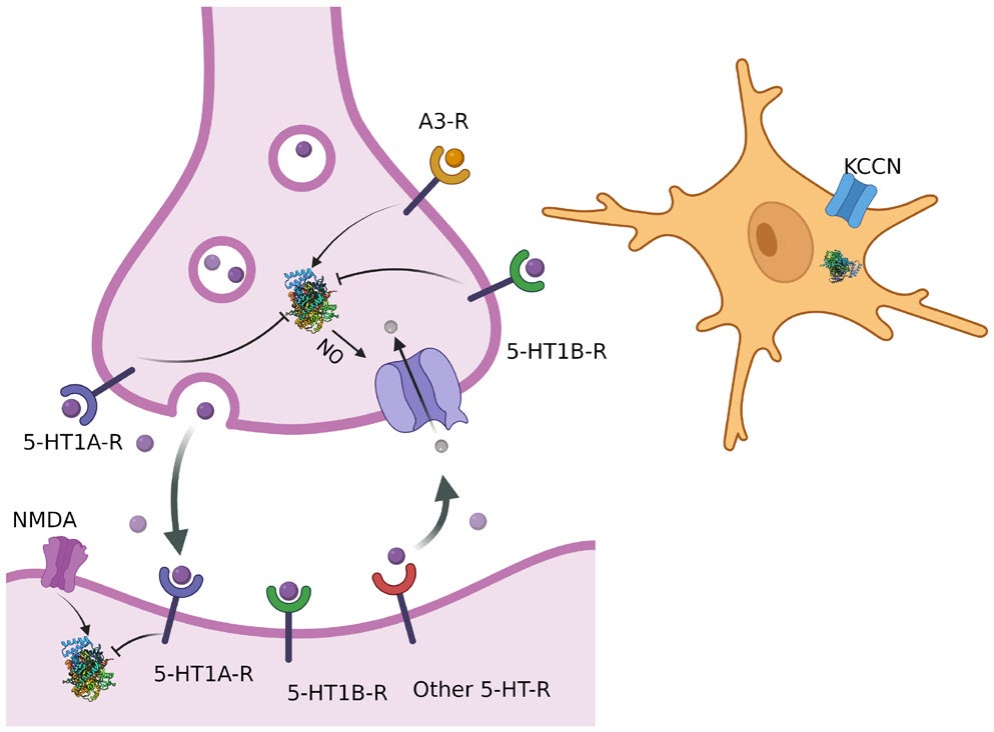
Interactions between nitric oxide (NO) and serotonergic signaling in neurons in vertebrates. Nitric oxide synthase (NOS) activity is regulated by activation of upstream signals, including the adenosine A3 receptors (A3‐R), whose activation stimulates NOS activity, and the serotonergic receptors 5‐HT1A‐R and 5‐HT1B‐R, whose activation inhibits NOS activity. NO then regulates serotonin transporter (SERT) activity, increasing reuptake. Thus, NO is both regulated by and regulates serotonergic signaling. Also shown is the opposing regulation of NOS activity by glutamatergic NMDA receptors and serotonergic 5‐HT1A receptors.

As observed above, in zebrafish, NO mediates the behavioral effects of drugs that act at different receptors (the 5‐HT1A and 5‐HT1B receptors, the adenosine A3 receptor, and the NMDA receptor). This suggests that, in serotonergic neurons, NO production is inhibited by the activation of 5‐HT1A and 5‐HT1B receptors, and increased by the activation of adenosine receptors and NMDA receptors (Figure 4). Moreover, in zebrafish, presynaptic NO modulates 5‐HT uptake through a cGMP‐dependent mechanism. Thus, serotonin levels at autoreceptors are able to dynamically modulate extracellular 5‐HT levels via the NO/cGMP/SERT cascade, tonically inhibiting it. This tonic inhibition of NO production in presynaptic sites is likely to be more relevant during tonic and low‐frequency phasic activities of serotonergic neurons, when serotonin is confined within synaptic clefts due to efficient retrieval by transporters, as shown in rodents (Zhang et al. 2025). In mice, during high‐frequency phasic serotonergic activity, excess serotonin surpasses transporter capacity, changing from synaptic to volume transmission (Zhang et al. 2025). Given the data found in zebrafish, it is plausible to suppose that this also occurs when low‐frequency activity is counterbalanced by phasic activation of adenosine and glutamate receptors. This tonic mode has been found to be important to regulate defensive behavior in zebrafish, representing an aversive expectation value via serotonergic projections to the telencephalon (Amo et al. 2014; Lima‐Maximino et al. 2020). It is also likely that activation of postsynaptic 5‐HT1A and 5‐HT1B receptors modulates the activity of targets of serotonergic projections in, e.g., limbic telencephalon, habenula, hypothalamus, or midbrain circuits to “fine‐tune” NO responses to phasic NMDA activation.

As discussed above, NO produced via NOS‐2 (“inducible”) proteins is also important in longer‐term regulation of defensive responses, especially those related to traumatic stressors. Activation of calcium‐activated potassium (KCNN) channels appears to be important in stress‐induced sensitization (de Sousa et al. 2024), a mechanism that might be independent of either 5‐HT, adenosine, or NMDA receptor activation. Thus, in zebrafish, NO appears to be a downstream mechanism that integrates responses from different receptors at the same time, contributing to the interplay between neurons and glial cells in the mediation of stress and defensive behavior.

## 6 Nitric Oxide, Stress, and Metaplasticity

“Metaplasticity” refers to the activity‐dependent preparation and priming of synapses for future change, shaping the direction, magnitude, and/or duration of plasticity‐related events (Abraham and Bear 1996; Hulme et al. 2013). While metaplasticity is activity‐dependent, and therefore induced by synaptic or cellular activity, it manifests as a change in the ability to induce subsequent synaptic plasticity by mechanisms such as long‐term potentiation (LTP) and long‐term depression (LDP), and not necessarily as observed changes in synaptic transmission efficacy (Abraham and Bear 1996). The balance between long‐term potentiation and long‐term depression is important in networks that mediate learning and memory; for example, too much potentiation could lead to either functional maladaptation or excitotoxicity, while too much depression can render networks nonresponsive. Thus, metaplasticity is an important mechanism by which synapses are prepared, including by setting threshold levels for depolarization and intracellular signaling, leading to the homeostatic regulation of the balance between stability and the persistent availability of plasticity (Hulme et al. 2013). These processes are impacted by stress; for example, rats exposed to a cat predator show increased LTP in a central amygdala–periaqueductal gray and a ventral angular bundle–basolateral amygdala pathways in comparison to unstressed animals (Adamec et al. 2006). Interestingly, the NMDA receptor antagonist CPP blocks this effect observed 9 days later in the right hemisphere, but potentiates it in the left hemisphere (Adamec et al. 2005).

The roles of NO in metaplasticity mechanisms have been described mainly in vitro. In CA1 slices, high‐frequency stimulation induces LTP, an effect that is dependent on NMDA receptors; however, activation of NMDA receptors either immediately prior to or following stimulation inhibits LTP generation (Zorumski and Izumi 1998). Treatment with NOS inhibitors overcomes this NMDA‐R‐dependent LTP inhibition (Izumi et al. 2008). p38 mitogen‐activated protein kinase (MAPK), an intracellular protein kinase that is also involved in the nitrergic mediation of serotonin uptake (Chang et al. 2012; Zhu et al. 2004), appears to be involved in this effect, as treatment with p38 inhibitors overcomes the LTP inhibition, but not inhibition of extracellular signal‐regulated kinase 1/2 (ERK1/2) or c‐Jun‐N‐terminal kinase (Izumi et al. 2008). p38 appears to be downstream of NO in this cascade, as treatment of slices with the NO donor SNP also blocks LTP, and this effect is overcome by p38 antagonists (Izumi et al. 2008). Photolytically released nitric oxide also inhibits LTP through a reduction in the efficacy of NMDA receptor function (Murphy and Bliss 1999). Moreover, weak tetanic stimulation, which does not usually induce LTP in hippocampal slices, is able to promote plasticity in the presence of NOS inhibitors, suggesting that NO can “fine‐tune” metaplasticity (Zorumski and Izumi 1998).

These mechanisms have not been directly tested in stress models, including zebrafish. However, multiple mediators of stress have been shown to induce metaplasticity, either increasing or decreasing the probability of LTP or LTD induction (Inoue et al. 2013; Karst et al. 2010; Maity et al. 2015). One hypothesis of how NO could mediate metaplasticity in situations of stress involves glutamate release from principal neurons from areas involved in defensive behavior, such as those outlined in Figure 2. During stress, the release of norepinephrine and circulating levels of corticosterone prepare defensive circuits for LTP induction, while low‐level NO production can decrease posterior responsiveness either by acting on GABAergic neurons or by inhibiting posterior LTP (Zorumski and Izumi 2012). The observation that NO has a biphasic effect on anxiety‐like behavior in zebrafish (Herculano et al. 2015) already suggested that this neurotransmitter could serve as a “dial” to fine‐tune stress‐responsive behaviors; its role in stress‐induced sensitization (de Sousa et al. 2024; Lima et al. 2015) and in the sensitization of defensive circuits after alcohol withdrawal (Chaves et al. 2020) also points to an important role of this molecule and the complex network of regulators and mediators in producing long‐term behavioral adjustments.

## 7 Conclusion

The biophysical properties of NO, its role as a downstream integrator of different upstream signals, and its role in both neuroplasticity and neurotoxicity make this gaseous transmitter an interesting target to understand the neurobehavioral mechanisms of stress. Zebrafish are no exception in this; as we have demonstrated in this review, although behavioral studies targeting NO signaling are still incipient, there is a great potential for this area of research. Outstanding questions still remain, however (Table 2). These questions await empirical responses and are reason enough to bet on the future importance of zebrafish in the field.

## Author Contributions

**João Alphonse Apóstolo Heymbeeck:** conceptualization, writing – original draft, visualization. **Eveline Bezerra de Sousa:** conceptualization, writing – review and editing. **Monica Lima‐Maximino:** conceptualization, writing – original draft, supervision, project administration. **Antonio Pereira Jr.:** conceptualization, visualization, writing – review and editing. **Caio Maximino:** conceptualization, writing – original draft.

## Conflicts of Interest

The authors declare no conflicts of interest.

## Peer Review

The peer review history for this article is available at https://www.webofscience.com/api/gateway/wos/peer‐review/10.1111/jnc.70254.

## Data Availability

Data sharing not applicable to this article as no datasets were generated or analysed during the current study.
